# Supplementary material for: Deep learning unlocks the true potential of organ donation after circulatory death with accurate prediction of time-to-death
Source: Sci Rep. 2025 Apr 19;15:13565. doi: 10.1038/s41598-025-95079-7 (PMC12009369; doi:10.1038/s41598-025-95079-7)
Supplement: Supplementary file 1 — Supplementary Information. [file 41598_2025_95079_MOESM1_ESM.pdf]

Supplementary Materials

Extending TTD Prediction to Longer Time Thresholds

In our study, we categorized time-to-death (TTD) into four intervals (0–30 min, 30–60 min, 60–120 min, and >120 min), following standard criteria and the extended spectrum for liver and kidney transplantation used by most U.S. transplant centers.

To demonstrate the flexibility of our model for potential application in other DCD protocols, such as international centers with longer time thresholds, we expanded the categories to six intervals (0–30 min, 30–60 min, 60–120 min, 120–180 min, 180–240 min, and >240 min). As a proof of concept, we trained and evaluated all methods in this extended setting (Table S1). ODE-RNN maintained high accuracy, low calibration error, and generally outperformed other models, demonstrating its adaptability to broader time scales.

Table S1. Performance comparison among various machine learning models in predicting extended TTD categories on the Yale New Haven Hospital (YNHH) test cohort and an external validation cohort. Metrics include accuracy, area under the receiver operating characteristic curve (ROC-AUC), area under the precision-recall curve (PR-AUC), and expected calibration error (ECE). Note that in 6-way classification, random chance corresponds to an accuracy of 0.167. Our model (ODE-RNN) demonstrates high accuracy, low calibration error, and generally outperforms other models.

model	UNOS	XGB	RNN	LSTM	GRU	GRU-D	ODE-RNN
Yale New Haven Hospital Test Cohort (Temporal Split, After 2021)							
Accuracy (6-way) ↑	0.420 ± 0.000	0.669 ± 0.000	0.746 ± 0.023	0.792 ± 0.006	**0.804** ± 0.010	0.780 ± 0.018	0.802 ± 0.007
Accuracy (<30 vs. >30) ↑	0.463 ± 0.000	0.900 ± 0.000	0.926 ± 0.016	0.943 ± 0.003	0.947 ± 0.006	0.941 ± 0.007	**0.955** ± 0.005
Accuracy (<60 vs. >60) ↑	0.521 ± 0.000	0.928 ± 0.000	0.890 ± 0.023	0.935 ± 0.005	0.949 ± 0.005	0.939 ± 0.005	**0.954** ± 0.006
Accuracy (<120 vs. >120) ↑	0.585 ± 0.000	0.934 ± 0.000	0.888 ± 0.026	0.910 ± 0.011	0.922 ± 0.014	0.907 ± 0.017	**0.939** ± 0.008
Accuracy (<180 vs. >180) ↑	0.612 ± 0.000	0.825 ± 0.000	0.892 ± 0.016	0.911 ± 0.009	**0.912** ± 0.007	0.900 ± 0.011	0.912 ± 0.005
Accuracy (<240 vs. >240) ↑	0.665 ± 0.000	0.855 ± 0.000	0.882 ± 0.008	**0.896** ± 0.012	0.896 ± 0.007	0.889 ± 0.007	0.884 ± 0.005
ROC-AUC (<30 vs. >30) ↑	0.581 ± 0.000	0.962 ± 0.000	0.952 ± 0.011	0.981 ± 0.007	0.975 ± 0.010	0.973 ± 0.009	**0.987** ± 0.002
ROC-AUC (<60 vs. >60) ↑	0.581 ± 0.000	0.966 ± 0.000	0.930 ± 0.012	0.971 ± 0.008	0.966 ± 0.008	0.964 ± 0.008	**0.986** ± 0.004
ROC-AUC (<120 vs. >120) ↑	0.608 ± 0.000	0.975 ± 0.000	0.923 ± 0.011	0.966 ± 0.008	0.958 ± 0.006	0.953 ± 0.009	**0.982** ± 0.005
ROC-AUC (<180 vs. >180) ↑	0.594 ± 0.000	0.526 ± 0.000	0.915 ± 0.008	0.955 ± 0.008	0.943 ± 0.006	0.938 ± 0.005	**0.965** ± 0.004
ROC-AUC (<240 vs. >240) ↑	0.600 ± 0.000	0.508 ± 0.000	0.891 ± 0.011	0.936 ± 0.010	0.919 ± 0.006	0.914 ± 0.005	**0.952** ± 0.005
PR-AUC (<30 vs. >30) ↑	0.598 ± 0.000	0.972 ± 0.000	0.958 ± 0.016	0.985 ± 0.006	0.975 ± 0.014	0.975 ± 0.008	**0.987** ± 0.001
PR-AUC (<60 vs. >60) ↑	0.662 ± 0.000	0.986 ± 0.000	0.958 ± 0.013	0.985 ± 0.005	0.978 ± 0.009	0.978 ± 0.007	**0.995** ± 0.001
PR-AUC (<120 vs. >120) ↑	0.747 ± 0.000	0.993 ± 0.000	0.966 ± 0.010	0.988 ± 0.006	0.981 ± 0.006	0.977 ± 0.008	**0.995** ± 0.001
PR-AUC (<180 vs. >180) ↑	0.790 ± 0.000	0.842 ± 0.000	0.973 ± 0.006	0.990 ± 0.002	0.983 ± 0.004	0.979 ± 0.007	**0.993** ± 0.001
PR-AUC (<240 vs. >240) ↑	0.821 ± 0.000	0.880 ± 0.000	0.972 ± 0.006	0.988 ± 0.002	0.981 ± 0.004	0.978 ± 0.006	**0.992** ± 0.001
ECE ↓	**0.021** ± 0.000	0.255 ± 0.000	0.059 ± 0.004	0.071 ± 0.014	0.059 ± 0.011	0.074 ± 0.014	0.034 ± 0.010
External Validation Cohort							
Accuracy (6-way) ↑	0.400 ± 0.000	0.667 ± 0.000	0.723 ± 0.021	0.750 ± 0.007	0.769 ± 0.011	0.751 ± 0.005	**0.784** ± 0.006
Accuracy (<30 vs. >30) ↑	0.434 ± 0.000	0.884 ± 0.000	0.915 ± 0.014	0.928 ± 0.005	0.941 ± 0.005	0.931 ± 0.005	**0.952** ± 0.004
Accuracy (<60 vs. >60) ↑	0.501 ± 0.000	0.898 ± 0.000	0.873 ± 0.016	0.913 ± 0.011	0.931 ± 0.005	0.918 ± 0.006	**0.952** ± 0.006
Accuracy (<120 vs. >120) ↑	0.555 ± 0.000	0.883 ± 0.000	0.852 ± 0.013	0.862 ± 0.011	0.886 ± 0.022	0.867 ± 0.014	**0.924** ± 0.005
Accuracy (<180 vs. >180) ↑	0.616 ± 0.000	0.837 ± 0.000	0.868 ± 0.018	0.880 ± 0.002	0.888 ± 0.007	0.884 ± 0.005	**0.904** ± 0.004
Accuracy (<240 vs. >240) ↑	0.659 ± 0.000	0.874 ± 0.000	0.873 ± 0.022	0.888 ± 0.009	0.890 ± 0.012	**0.893** ± 0.003	0.885 ± 0.002
ROC-AUC (<30 vs. >30) ↑	0.518 ± 0.000	0.943 ± 0.000	0.947 ± 0.009	0.966 ± 0.002	0.968 ± 0.006	0.965 ± 0.003	**0.991** ± 0.002
ROC-AUC (<60 vs. >60) ↑	0.550 ± 0.000	0.952 ± 0.000	0.924 ± 0.011	0.951 ± 0.004	0.957 ± 0.005	0.951 ± 0.004	**0.986** ± 0.002
ROC-AUC (<120 vs. >120) ↑	0.531 ± 0.000	0.945 ± 0.000	0.888 ± 0.010	0.918 ± 0.008	0.929 ± 0.004	0.923 ± 0.008	**0.972** ± 0.003
ROC-AUC (<180 vs. >180) ↑	0.568 ± 0.000	0.532 ± 0.000	0.869 ± 0.012	0.895 ± 0.010	0.905 ± 0.005	0.898 ± 0.009	**0.957** ± 0.002
ROC-AUC (<240 vs. >240) ↑	0.578 ± 0.000	0.534 ± 0.000	0.858 ± 0.011	0.879 ± 0.010	0.891 ± 0.006	0.885 ± 0.007	**0.941** ± 0.002
PR-AUC (<30 vs. >30) ↑	0.580 ± 0.000	0.961 ± 0.000	0.953 ± 0.011	0.973 ± 0.005	0.965 ± 0.013	0.968 ± 0.006	**0.995** ± 0.001
PR-AUC (<60 vs. >60) ↑	0.667 ± 0.000	0.978 ± 0.000	0.949 ± 0.012	0.973 ± 0.005	0.964 ± 0.011	0.966 ± 0.008	**0.995** ± 0.001
PR-AUC (<120 vs. >120) ↑	0.720 ± 0.000	0.985 ± 0.000	0.942 ± 0.009	0.969 ± 0.006	0.965 ± 0.004	0.964 ± 0.006	**0.993** ± 0.001
PR-AUC (<180 vs. >180) ↑	0.794 ± 0.000	0.879 ± 0.000	0.945 ± 0.005	0.972 ± 0.005	0.968 ± 0.003	0.967 ± 0.006	**0.992** ± 0.000
PR-AUC (<240 vs. >240) ↑	0.837 ± 0.000	0.907 ± 0.000	0.956 ± 0.004	0.975 ± 0.004	0.974 ± 0.003	0.973 ± 0.004	**0.992** ± 0.000
ECE ↓	0.100 ± 0.000	0.230 ± 0.000	0.056 ± 0.011	0.098 ± 0.012	0.077 ± 0.018	0.092 ± 0.011	**0.033** ± 0.008

Predicting TTD 12 Hours Before Extubation

While the up-to-date model that accumulates input variables until time of extubation yields the highest performance, it is also beneficial to have a dedicated model that predicts TTD several hours before extubation. This is particularly useful in real-world scenarios, as it allows the OPO to deploy the retrieval team in advance.

As shown in Table S2, we trained and evaluated all methods on early prediction of TTD using only historical data available up to 12 hours before extubation. The proposed ODE-RNN model remains the most competitive in this setting as well.

Table S2. Performance comparison among various machine learning models in predicting extended TTD categories (0–30 min, 30–60 min, 60–120 min, 120–180 min, 180–240 min, and >240 min) on the Yale New Haven Hospital (YNHH) test cohort and an external validation cohort. The models **only use data up to 12 hours before extubation**, to simulate early prediction of TTD. Metrics include the area under the receiver operating characteristic curve (ROC-AUC), area under the precision-recall curve (PR-AUC), and expected calibration error (ECE). Note that in 6-way classification, random chance corresponds to an accuracy of 0.167. Our model (ODE-RNN) demonstrates high accuracy, low calibration error, and generally outperforms other models.

model	UNOS	XGB	RNN	LSTM	GRU	GRU-D	ODE-RNN
Yale New Haven Hospital Test Cohort (Temporal Split, After 2021)							
Accuracy (6-way) ↑	0.410 ± 0.000	0.451 ± 0.000	0.483 ± 0.012	0.534 ± 0.032	0.539 ± 0.013	0.527 ± 0.019	**0.570** ± 0.028
Accuracy (<30 vs. >30) ↑	0.452 ± 0.000	0.845 ± 0.000	0.893 ± 0.021	0.928 ± 0.017	**0.936** ± 0.016	0.920 ± 0.014	0.906 ± 0.028
Accuracy (<60 vs. >60) ↑	0.511 ± 0.000	0.812 ± 0.000	0.790 ± 0.008	0.840 ± 0.014	0.853 ± 0.004	0.832 ± 0.024	**0.892** ± 0.016
Accuracy (<120 vs. >120) ↑	0.574 ± 0.000	0.793 ± 0.000	0.769 ± 0.021	0.790 ± 0.041	0.796 ± 0.021	0.783 ± 0.017	**0.891** ± 0.006
Accuracy (<180 vs. >180) ↑	0.601 ± 0.000	0.789 ± 0.000	0.757 ± 0.025	0.784 ± 0.025	0.777 ± 0.009	0.761 ± 0.012	**0.805** ± 0.012
Accuracy (<240 vs. >240) ↑	0.654 ± 0.000	**0.765** ± 0.000	0.716 ± 0.023	0.749 ± 0.013	0.752 ± 0.019	0.725 ± 0.012	0.749 ± 0.015
ROC-AUC (<30 vs. >30) ↑	0.591 ± 0.000	0.882 ± 0.000	0.911 ± 0.007	0.954 ± 0.007	**0.968** ± 0.011	0.954 ± 0.010	0.959 ± 0.010
ROC-AUC (<60 vs. >60) ↑	0.592 ± 0.000	0.871 ± 0.000	0.841 ± 0.011	0.908 ± 0.009	0.921 ± 0.013	0.894 ± 0.011	**0.931** ± 0.021
ROC-AUC (<120 vs. >120) ↑	0.610 ± 0.000	0.903 ± 0.000	0.834 ± 0.017	0.892 ± 0.010	0.904 ± 0.009	0.863 ± 0.012	**0.930** ± 0.028
ROC-AUC (<180 vs. >180) ↑	0.596 ± 0.000	0.887 ± 0.000	0.819 ± 0.011	0.864 ± 0.005	0.866 ± 0.008	0.837 ± 0.013	**0.896** ± 0.008
ROC-AUC (<240 vs. >240) ↑	0.603 ± 0.000	0.856 ± 0.000	0.776 ± 0.011	0.823 ± 0.005	0.821 ± 0.010	0.788 ± 0.016	**0.872** ± 0.015
PR-AUC (<30 vs. >30) ↑	0.604 ± 0.000	0.546 ± 0.000	0.633 ± 0.035	0.803 ± 0.050	**0.853** ± 0.060	0.786 ± 0.045	0.817 ± 0.051
PR-AUC (<60 vs. >60) ↑	0.669 ± 0.000	0.755 ± 0.000	0.696 ± 0.051	0.840 ± 0.023	0.858 ± 0.027	0.799 ± 0.035	**0.861** ± 0.044
PR-AUC (<120 vs. >120) ↑	0.747 ± 0.000	0.906 ± 0.000	0.800 ± 0.047	0.899 ± 0.012	0.900 ± 0.014	0.849 ± 0.022	**0.946** ± 0.018
PR-AUC (<180 vs. >180) ↑	0.791 ± 0.000	0.929 ± 0.000	0.857 ± 0.022	0.909 ± 0.005	0.906 ± 0.009	0.874 ± 0.016	**0.937** ± 0.013
PR-AUC (<240 vs. >240) ↑	0.822 ± 0.000	0.926 ± 0.000	0.858 ± 0.019	0.907 ± 0.005	0.902 ± 0.008	0.872 ± 0.015	**0.936** ± 0.016
ECE ↓	**0.030** ± 0.000	0.294 ± 0.000	0.069 ± 0.021	0.109 ± 0.003	0.104 ± 0.022	0.092 ± 0.014	0.053 ± 0.012
External Validation Cohort							
Accuracy (6-way) ↑	0.425 ± 0.000	0.369 ± 0.000	0.438 ± 0.015	0.500 ± 0.017	0.531 ± 0.022	0.490 ± 0.011	**0.547** ± 0.024
Accuracy (<30 vs. >30) ↑	0.456 ± 0.000	0.817 ± 0.000	0.891 ± 0.008	0.911 ± 0.007	**0.913** ± 0.004	0.904 ± 0.011	0.902 ± 0.029
Accuracy (<60 vs. >60) ↑	0.523 ± 0.000	0.765 ± 0.000	0.789 ± 0.025	0.871 ± 0.008	0.889 ± 0.011	0.866 ± 0.010	**0.894** ± 0.027
Accuracy (<120 vs. >120) ↑	0.579 ± 0.000	0.686 ± 0.000	0.696 ± 0.014	0.751 ± 0.033	0.796 ± 0.029	0.732 ± 0.021	**0.889** ± 0.013
Accuracy (<180 vs. >180) ↑	0.643 ± 0.000	0.733 ± 0.000	0.716 ± 0.009	0.746 ± 0.005	0.762 ± 0.008	0.730 ± 0.007	**0.822** ± 0.018
Accuracy (<240 vs. >240) ↑	0.687 ± 0.000	0.740 ± 0.000	0.710 ± 0.011	0.737 ± 0.014	0.752 ± 0.014	0.718 ± 0.011	**0.759** ± 0.021
ROC-AUC (<30 vs. >30) ↑	0.520 ± 0.000	0.843 ± 0.000	0.913 ± 0.004	0.953 ± 0.008	0.961 ± 0.007	0.953 ± 0.006	**0.970** ± 0.012
ROC-AUC (<60 vs. >60) ↑	0.549 ± 0.000	0.846 ± 0.000	0.852 ± 0.008	0.919 ± 0.005	0.937 ± 0.005	0.917 ± 0.010	**0.962** ± 0.007
ROC-AUC (<120 vs. >120) ↑	0.530 ± 0.000	0.809 ± 0.000	0.786 ± 0.007	0.857 ± 0.007	0.882 ± 0.004	0.833 ± 0.010	**0.933** ± 0.008
ROC-AUC (<180 vs. >180) ↑	0.567 ± 0.000	0.816 ± 0.000	0.765 ± 0.007	0.816 ± 0.008	0.830 ± 0.001	0.790 ± 0.008	**0.894** ± 0.008
ROC-AUC (<240 vs. >240) ↑	0.577 ± 0.000	0.797 ± 0.000	0.749 ± 0.008	0.793 ± 0.004	0.803 ± 0.004	0.767 ± 0.008	**0.858** ± 0.008
PR-AUC (<30 vs. >30) ↑	0.581 ± 0.000	0.574 ± 0.000	0.702 ± 0.043	0.755 ± 0.041	0.768 ± 0.068	0.744 ± 0.034	**0.827** ± 0.059
PR-AUC (<60 vs. >60) ↑	0.667 ± 0.000	0.749 ± 0.000	0.743 ± 0.021	0.841 ± 0.017	0.830 ± 0.025	0.806 ± 0.016	**0.943** ± 0.013
PR-AUC (<120 vs. >120) ↑	0.720 ± 0.000	0.833 ± 0.000	0.760 ± 0.015	0.860 ± 0.017	0.840 ± 0.012	0.805 ± 0.017	**0.952** ± 0.004
PR-AUC (<180 vs. >180) ↑	0.793 ± 0.000	0.887 ± 0.000	0.801 ± 0.011	0.875 ± 0.012	0.850 ± 0.009	0.829 ± 0.013	**0.947** ± 0.003
PR-AUC (<240 vs. >240) ↑	0.837 ± 0.000	0.911 ± 0.000	0.844 ± 0.007	0.898 ± 0.007	0.880 ± 0.005	0.864 ± 0.010	**0.946** ± 0.002
ECE ↓	0.076 ± 0.000	0.310 ± 0.000	0.070 ± 0.012	0.128 ± 0.018	0.092 ± 0.011	0.108 ± 0.016	**0.052** ± 0.020

Calibration plots

In addition to the calibration plots for TTD < 30 min vs. > 30 min shown in Figure 2 panel B, we present the same analyses for 60-minute and 120-minute marks.

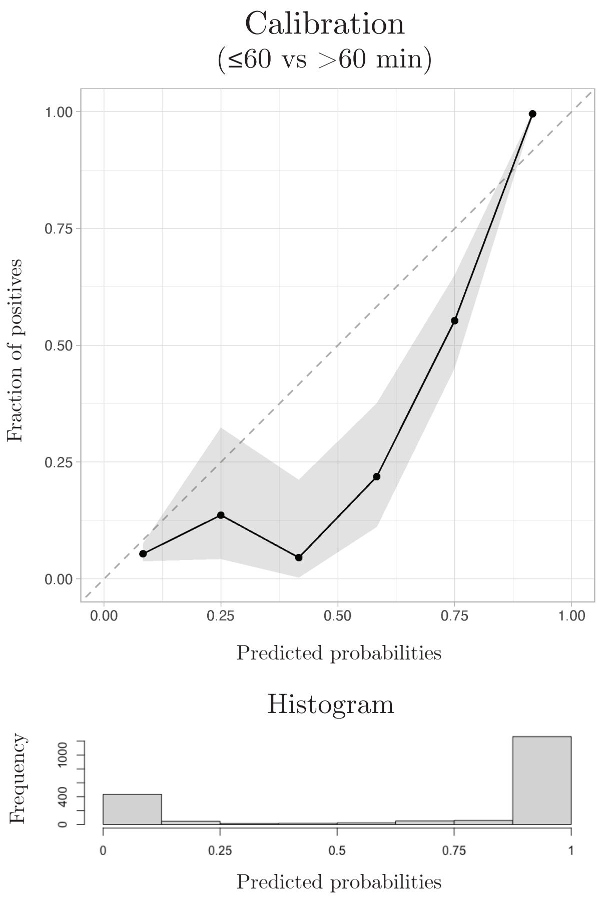

Figure S1. Calibration plot for the binary classification task TTD <60 min vs. TTD >60 min on the external validation cohort, computed with the R package val.prob.ci.2. The predicted probabilities come from the output of our model and plotted against the fraction of positives observed in the data. The histogram shows the prevalence of patients for different ranges of predicted probabilities.

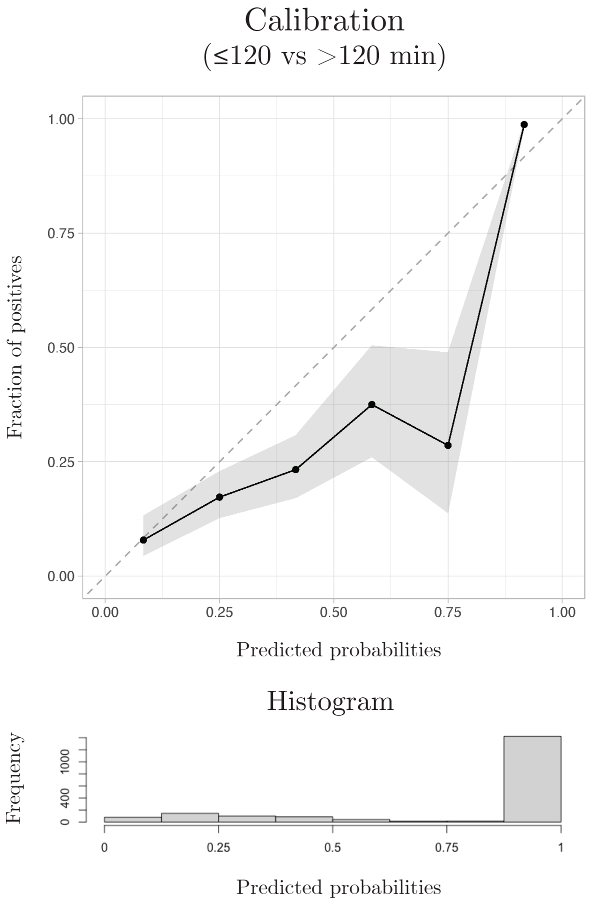

Figure S2. Calibration plot for the binary classification task TTD <120 min vs. TTD >120 min on the external validation cohort, computed with the R package val.prob.ci.2. The predicted probabilities come from the output of our model and plotted against the fraction of positives observed in the data. The histogram shows the prevalence of patients for different ranges of predicted probabilities.
